# Emergence of Swarming Behavior: Foraging Agents Evolve Collective Motion Based on Signaling

**DOI:** 10.1371/journal.pone.0152756

**Published:** 2016-04-27

**Authors:** Olaf Witkowski, Takashi Ikegami

**Affiliations:** 1 Department of Interdisciplinary Studies, Graduate School of Arts and Sciences, The University of Tokyo, 3-8-1 Komaba, Meguro-ku 153-8902 Tokyo, Japan; 2 ELSI Origins Network, Earth-Life Science Institute, Tokyo Institute of Technology, 2-12-1-IE-1 Ookayama, Meguro-ku 152-8550 Tokyo, Japan; National Scientific and Technical Research Council (CONICET)., ARGENTINA

## Abstract

Swarming behavior is common in biology, from cell colonies to insect swarms and bird flocks. However, the conditions leading to the emergence of such behavior are still subject to research. Since Reynolds’ boids, many artificial models have reproduced swarming behavior, focusing on details ranging from obstacle avoidance to the introduction of fixed leaders. This paper presents a model of evolved artificial agents, able to develop swarming using only their ability to listen to each other’s signals. The model simulates a population of agents looking for a vital resource they cannot directly detect, in a 3D environment. Instead of a centralized algorithm, each agent is controlled by an artificial neural network, whose weights are encoded in a genotype and adapted by an original asynchronous genetic algorithm. The results demonstrate that agents progressively evolve the ability to use the information exchanged between each other via signaling to establish temporary leader-follower relations. These relations allow agents to form swarming patterns, emerging as a transient behavior that improves the agents’ ability to forage for the resource. Once they have acquired the ability to swarm, the individuals are able to outperform the non-swarmers at finding the resource. The population hence reaches a neutral evolutionary space which leads to a genetic drift of the genotypes. This reductionist approach to signal-based swarming not only contributes to shed light on the minimal conditions for the evolution of a swarming behavior, but also more generally it exemplifies the effect communication can have on optimal search patterns in collective groups of individuals.

## Introduction

The ability of fish schools, insect swarms or starling murmurations to shift shape as one and coordinate their motion in space has been studied extensively because of their implications for the evolution of social cognition, collective animal behavior and artificial life [1–6].

Swarming is the phenomenon in which a large number of individuals organize into a coordinated motion. Using only the information at their disposition in the environment, they are able to aggregate together, move *en masse* or migrate towards a common direction.

The movement itself may differ from species to species. For example, fish and insects swarm in three dimensions, whereas herds of sheep move only in two dimensions. Moreover, the collective motion can have quite diverse dynamics. While birds tend to flock in relatively ordered formations with constant velocity, fish schools change directions by aligning rapidly and keeping their distances, and most insects swarms (with perhaps exceptions such as locusts [7]) move in a messy and random-looking way [8–10].

Numerous evolutionary hypotheses have been proposed to explain swarming behavior across species. These include more efficient mating, good environment for learning, combined search for food resources, and reducing risks of predation [11]. Pitcher and Partridge [12] also mention energy saving in fish schools by reducing drag.

In an effort to test the multiple theories, the past decades counted several experiments involving real animals, either inside an experimental setup [13–15] or observed in their own ecological environment [16]. Those experiments present the inconvenience to be costly to reproduce. Furthermore, the colossal lapse of evolutionary time needed to evolve swarming makes it almost impossible to study the emergence of such behavior experimentally.

Computer modeling has recently provided researchers with new, easier ways to test hypotheses on collective behavior. Simulating individuals on machines offers easy modification of setup conditions and parameters, tremendous data generation, full reproducibility of every experiment, and easier identification of the underlying dynamics of complex phenomena.

### From Reynolds’ boids to recent approaches

Reynolds [17] introduces the *boids* model simulating 3D swarming of agents called *boids* controlled only by three simple rules:

- *Alignment*: move in the same direction as neighbours
- *Cohesion*: remain close to neighbours
- *Separation*: avoid collisions with neighbours

These rules can be translated into differential equations based on the velocity of each boid:

$$\begin{array}{c} \vec{\Delta v_{i}}=w_{c}\cdot \left(\vec{r_{i}}-\frac{\sum_{j\in S_{c}}\vec{r_{j}}}{n_{c}}\right)+w_{a}\cdot \left(\vec{v_{i}}-\frac{\sum_{j\in S_{a}}\vec{v_{j}}}{n_{a}}\right)+w_{s}\cdot \left(\sum_{j\in S_{s}}\frac{\left(\vec{r_{i}}-\vec{r_{j}}\right)}{\left|(\vec{r_{i}}-\vec{r_{j}})\right|}\right) \end{array}$$ (1)

The position of each boid is updated by the computed velocity $\vec{\Delta r_{i}}$ iteratively. The attraction and repulsion terms are represented by the first and second term, respectively. Each rule has an interaction range around each agent and is respectively denoted by *S*_*c*_, *S*_*s*_ and *S*_*a*_. In the equation, the amplitudes of those interactions are respectively *w*_*c*_, *w*_*s*_, and *w*_*a*_, and the speed amplitude is typically bounded between values *v*_*min*_ and *v*_*max*_.

Various works have since then reproduced swarming behavior, often by the means of an explicitly coded set of rules. For instance, Mataric [18] proposes a generalization of Reynolds’ original model with an optimally weighted combination of six basic interaction primitives (namely, collision avoidance, following, dispersion, aggregation, homing and flocking). Vicsek [6] models the swarming of point particles, moving at a constant speed, in the average direction of motion of the local neighbors with some added noise. Hartman and Benes [19] come up with yet another variant of the original model, by adding a complementary force to the *alignment* rule, that they call *change of leadership*. Many other approaches have been based on informed agents or fixed leaders [20–22]. Unfortunately, in spite of the insight this kind of approach brings into the dynamics of swarming, it shows little about the pressures leading to its emergence.

For that reason, experimenters attempted to simulate swarming without a fixed set of rules, rather by incorporating into each agent an artificial neural network brain that controls its movements, namely using the evolutionary robotics approach. The swarming behavior is evolved by copy with mutations of the chromosomes encoding the neural network parameters. By comparing the impact of different selective pressures, this type of methodology, first used in [23] to solve optimization problems, eventually allowed to study the evolutionary emergence of swarming.

Tu and Terzopoulos [24] have swarming emerge from the application of artificial pressures consisting of hunger, libido and fear. Other experimenters have analyzed prey/predator systems to show the importance of sensory system and predator confusion in the evolution of swarming in preys [25, 26].

In spite of many pressures hypothesized to produce swarming behavior, designed setups presented in the literature are often complex and specific. Previous works typically introduce models with very specific environments, where agents are designed to be more sensitive to particular inputs. While they are bringing valuable results to the community, one may wonder about with a more general, simpler design.

Recently, studies such as in Torney et al. [27] successfully showed the advantages of signaling to climb environmental gradients. However, these models hard-code the fact that individuals turn towards each other based on the signals they emit, unlike the evolutionary robotics approach mentioned previously which attempts to have the swarming behavior evolve. Collective navigation has also been shown to allow for a dampening of the stochastic effects of individual sampling errors, helping groups climb gradients [28, 29]. This effect has also been highlighted for migration [30, 31].

Finally, even when studies focus on fish or insects, which swarm in 3D [26], most keep their model in 2D. While swarming is usually robust against dimensionality change, the coding for such behavior from 2D to 3D has been shown to require a non-trivial mapping, in the sense that the dependence on parameters can vary case by case [32]. Indeed, the addition of a third degree of freedom may enable agents to produce significantly distinct and more complex behaviors.

### Signaling agents in a resource finding task

This paper studies the emergence of swarming in a population of agents using a basic signaling system, while performing a simple resource gathering task.

Simulated agents move around in a three dimensional space, looking for a vital but invisible food resource randomly distributed in the environment. The agents are emitting signals that can be perceived by other individuals’ sensors within a certain radius. Both agent’s motion and signaling are controlled by an artificial neural network embedded in each agent, evolved over time by an asynchronous genetic algorithm. Agents that consume enough food are enabled to reproduce, whereas those whose energy drops to zero are removed from the simulation.

Each experiment is performed in two steps: training the agents in an environment with resource locations providing fitness, then testing in an environment without fitness.

During the training, we observe that the agents progressively come to coordinate into clustered formations. That behavior is then preserved in the second step. Such patterns do not appear in control experiments having the simulation start directly from the second phase, with the absence of resource locations. This means that the presence of the resource is needed to make the clustered swarming behavior appear. If at any point the signaling is switched off, the agents immediately break the swarming formation. A swarming behavior is only observed once the communication is turned back on. Furthermore, the simulations with signaling lead to agents gathering very closely around food patches, whereas control simulations with silenced agents end up with all individuals wandering around erratically.

The main contribution of this work is to show that collective motion can originate, without explicit central coordination, from the combination of a generic communication system and a simple resource gathering task. As a secondary contribution, our model also demonstrates how swarming behavior, in the context of an asynchronous evolutionary simulation, can lead to a neutral evolutionary space, where no more selection is applied on the gene pool.

A specific genetic algorithm with an asynchronous reproduction scheme is developed and used to evolve the agents’ neural controllers. In addition, the search for resource is shown to improve from the agents clustering, eventually leading to the agents gathering closely around goal areas. An in-depth analysis shows increasing information transfer between agents throughout the learning phase, and the development of leader/follower relations that eventually push the agents to organize into clustered formations.

## Methods

### Agents in a 3D world

We simulate a group of agents moving around in a continuous, toroidal arena of 600.0 × 600.0 × 600.0 (in arbitrary units). Each agent is characterized by the internal neural network (i.e. neural states *x*_*i*_ and the connections among them *w*_*ij*_), 6 inputs *I*_*i*_ (i = 1, 2, … , 6) and 3 outputs *O*_*i*_ (i = 1, 2, 3) computed from the inputs through the neural network.

The agents have energy which is consumed by their moving around. If at any point an agent’s energy drops to zero, the agent is dead and is immediately removed from the environment.

The agent’s position is determined by three floating point coordinates between 0.0 and 600.0. Each agent is positioned randomly at the start of the simulation, and then moves at a fixed speed of 1.0 arbitrary unit per iteration.

The navigation schema of each agent consists of 3 steps.

**Fig 1:**
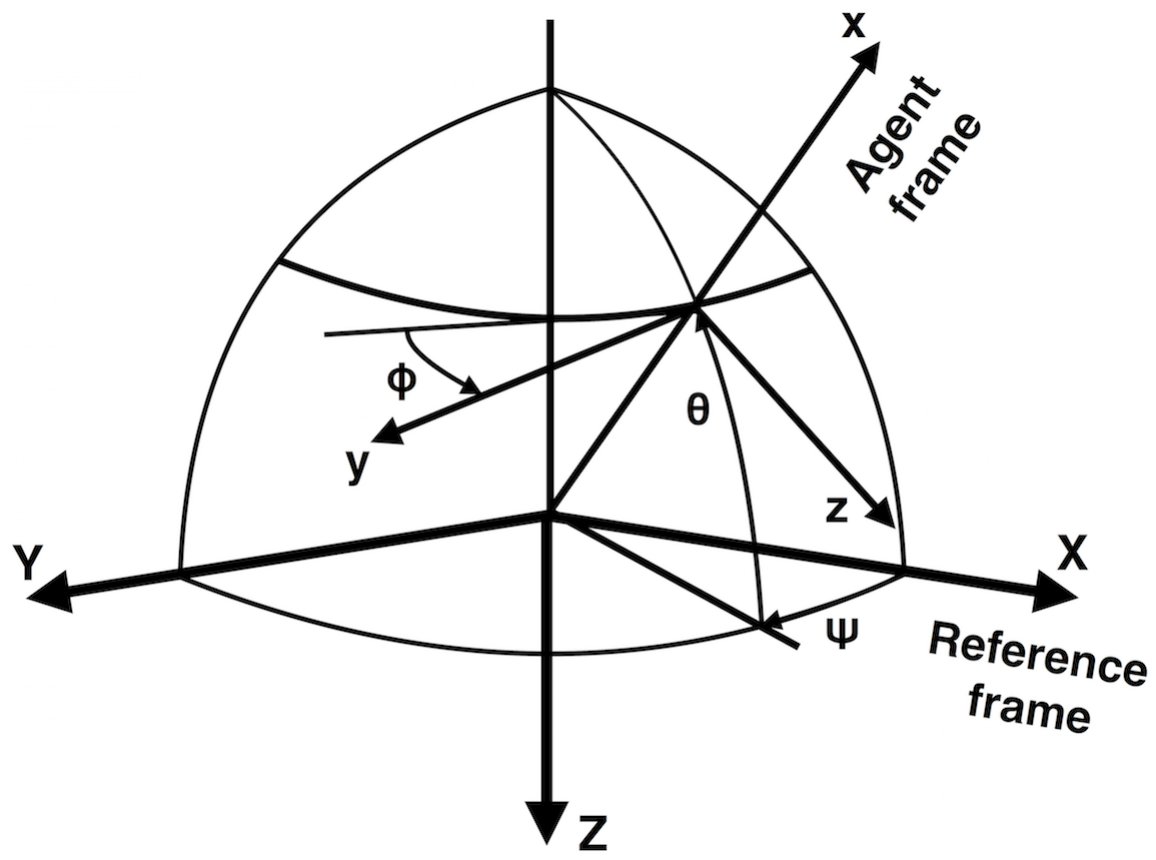
Illustration of Euler angles. *ψ* corresponds to the agent’s pitch (i.e. elevation) and *θ* is the agent’s yaw (i.e. heading). The agent’s roll *ϕ* is not used in this paper.

1. The agent’s velocity ${\vec{v}}_{i}\left(t\right)$ is updated by the following equation;
  $$\begin{array}{c} \vec{v_{i}}\left(t\right)=\vec{v_{i}}\left(t-1\right)+c_{1}\cdot tan\left(\theta \right)\cdot \vec{n}+c_{2}\cdot tan\left(\psi \right)\cdot \vec{v_{i}}\left(t-1\right) \end{array}$$ (2)
  from the two Euler angles (*ψ* for the agent’s pitch (i.e. elevation) and *θ* for the agent’s yaw (i.e. heading)) at the previous time step. Fig 1 illustrates the Euler angles, as they are used in our model.
2. The angles are updated as follows:
  $$\begin{array}{c} \psi =2c\tan^{-1}\frac{z}{x}\psi =2c\sin^{-1}\frac{y}{\left||(x,y,z)|\right|} \end{array}$$ (3)
  while the norm of the velocity $∥{\vec{v}}_{i}∥$ is kept constant (like in [6]).
3. The position of agent i ${\vec{x}}_{t}$ is then updated according to its current velocity with, instead of Eq 1:
  $$\begin{array}{c} \vec{r_{i}}\left(t\right)=\vec{r_{i}}\left(t-1\right)+\vec{v_{i}}\left(t\right) \end{array}$$ (4)

We iterate the three steps in order to determine the next positions and velocities of each agent. In order to compute the second step above, we need to calculate the outputs of neural networks.

### Agents controlled by neural networks

A neural network consists of 6 sensors, a fully connected 10-neurons hidden layer and 3 output neurons that encode two motor outputs and one which produces the communication signal. Each sensor and output state takes continuous values between 0 and 1, but the output states are converted into two Euler angles (see in the previous section) and one communication signal.

Each activation state *y*_*i*_ of a neuron *i* takes a value in the interval between 0 and 1 and is updated according to:

$$\begin{array}{c} y_{i}=\sigma \left(\sum_{j}^{n}w_{ji}y_{j}\right) \end{array}$$ (5)

where *w*_*ji*_ the weight from neuron *j* to neuron *i*, and *σ* is the sigmoid function defined as:

$$\begin{array}{c} \sigma \left(x\right)=\frac{1}{1+e^{-\beta x}} \end{array}$$ (6)

with *β* the slope parameter.

**Fig 2:**
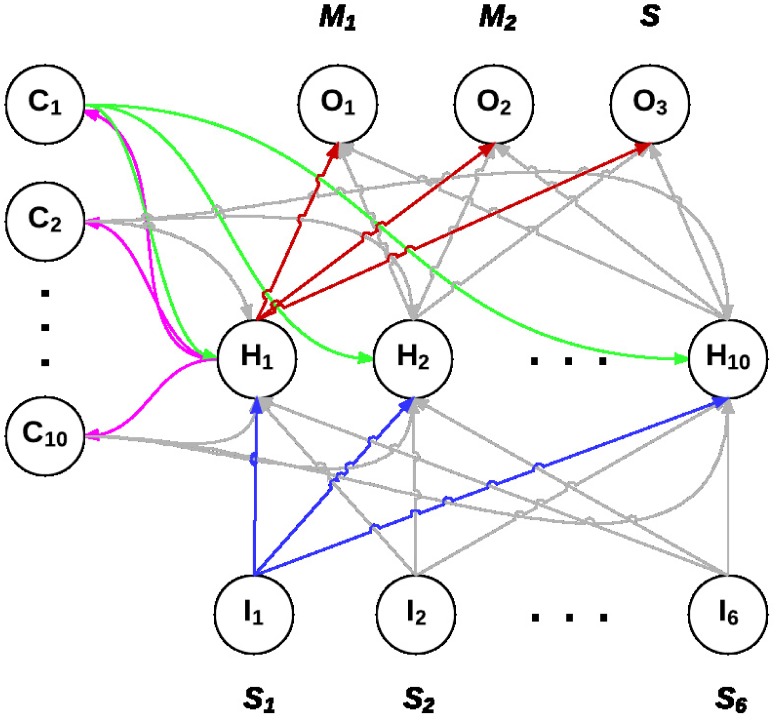
Architecture of the agent’s controller, a recursive neural network composed of 6 input neurons (*I*_1_ to *I*_6_), 10 hidden neurons (*H*_1_ to *H*_10_), 10 context neurons (*C*_1_ to *C*_10_) and 3 output neurons (*O*_1_ to *O*_3_). The input neurons receive signal values from neighboring agents, with each neuron corresponding to signals received from one of the 6 sectors in space. The output neurons *O*_1_ and *O*_2_ control the agent’s motion, and *O*_3_ controls the signal it emits. The context neurons have connections from and to the hidden layer, thus creating a feedback allowing for a state maintenance effect.

The connections between neurons are defined according to the architecture shown in Fig 2. Each connection’s weight *w*_*ji*_ in the neural network takes continuous values between 0 and 1. The connection weights are put into a gene string which constitutes the agent’s genotype, and is then evolved using a specific genetic algorithm described below.

### Communication among agents

Every agent is capable of sending signals with intensities (signals are encoded as floating point values ranging from 0.0 to 1.0). One of three outputs, *O*_3_, is assigned as a signal.

Six input sensors (*I*_*i*_ (i = 1, 2, … , 6)) of each agent are to detect signals produced by other agents. The sensors are put on the front, rear, left, right, top and bottom of the agent’s spherical body and collect inputs up to a distance of 100 units from 6 directions, respectively.

The distance to the source proportionally affects the intensity of a received signal, and signals from agents above a 100-distance are ignored. The sensor whose direction is the closest to the signaling source receives one float value, equal to the sum of every signal emitted within range, divided by the distance, and normalized between 0 and 1.

### Genetic algorithm and an asynchronous reproduction scheme

Genetic algorithms [33–35] simulate the descent with modification of a population of chromosomes, selected generation after generation by a defined fitness function.

Our model differs from the usual genetic algorithm paradigm, in that it designs variation and selection in an asynchronous way, similarly to steady state genetic algorithms in [36, 37]. The reproduction takes place continuously throughout the simulation, creating overlapping generations of agents. This allows for a more natural, continuous model, as no global clock is defined that could bias the results.

Practically we iterate the following evolution dynamics in this order:

1. Every new agent is born with an energy equal to 2.0.
2. Each agent can lose a variable amount of energy depending on the behavior during a given period. More precisely, agents spend a fixed amount of energy for movement *C*_*mov*_ (0.01 per iteration) and a variable amount of energy *C*_*sig*_ for signaling costs (0.001 ⋅ *I*_*sig*_ per iteration where *I*_*sig*_ is the signal intensity).
3. Each agent can gain at time *t* from an energy source by $R_{i}\left(t\right)=\frac{r}{d_{i}\left(t\right)}$ where *r* is the reward value and *d*_*i*_ is the agent’s distance to the center of the energy source. The reward falls off inversely proportionally to *d*.
4. A total sum of energy *F*_*i*_ is computed, which is adjusted such that the population size remains between 150 and 250 agents. This adjustement is given by introducing the cost factor *c* as follows: *F*_*i*_(*t*) = *R*_*i*_−*c* ⋅ (*C*_*mov*_(*t*)−*C*_*sig*_(*t*)).
5. Whenever an agent accumulates 10.0 in its energy value, a replica of itself with a 5% mutation in the genotype is created and added to a random position in the arena (the choice for random initial positions is to avoid biasing the proximity of agents, so that reproduction does not become a way for agents to create local clusters). Each mutation changes the value of an allele by an offset value picked uniformly at random between −0.05 and 0.05. The agent’s energy is decreased by 8.0 and the new replica’s energy is set to 2.0.
6. Every 5000 iterations, the energy source site is randomly reassigned.

There are a few remarks in running this evolution schema. First, a local reproduction scheme (i.e. giving birth to offspring close to their parents) leads rapidly to a burst in population size (along with an evolutionary radiation of the genotypes), as the agents that are close to the resource create many offspring that will be very fit too, thus able to replicate very fast as well. This is why we choose to introduce newborn offspring randomly in the environment. On a side note, population bursts occur solely when the neighborhood radius is small (under 10), while values over 100 do not lead to population bursts.

Second, for the genetic algorithm to be effective, the number of agents must be maintained above a certain level. Also, the computation power limits the population size.

Third, the energy value allowed to the agents is therefore adjusted in order to maintain an ideal number as close as possible to 200 (and always comprised between 50 and 1000, passed which there is respectively no removal or reproduction of agents allowed) agents alive throughout the simulation. The way it is done in practice is by adjusting the energy cost of every agent by a multiplying factor 1.0001 if the population is higher than 250 individuals and a divide it by 1.01 if the population is lower than 150.

Finally, the agents above a certain age (5000 time steps) are removed from the simulation, to keep the evolution moving at an adequate pace.

### Experimental setup

Additional to the above evolution schema, we executed the simulation in two steps: training and testing: In the training step, the resource locations are randomly distributed over the environment space.

In the testing step, the fitness function is ignored, and the resource is simply distributed equally among all the agents, meaning that they all receive an equal share of resource sufficient to keep them alive. As a consequence, the agents do not die off, and that second step conserves the same population of individuals, in order to test their behavior. From this point, whenever not mentioned otherwise, the analyses are referring to the first step, during which the swarming behavior comes about progressively. The purpose of the second step of the experiment is uniquely aimed at checking the behavior of the resulting population of agents without resources, that is to say, without reproduction, as the cost in energy is controlled to maintain the population in a reasonable interval.

The parameter values used in the simulations are detailed in Table 1.

**Table 1:** Summary of the simulation parameters.

Parameter	Value
Initial/average number of agents	200
Maximum number of agents	1000
Minimum number of agents	100
Agent maximum age (iterations)	5000
Maximum agent energy	100
Maximum energy absorption (per iteration)	1
Maximum neighborhood radius	100
Map dimensions (side of the cube)	600
Reproduction radius	10
Initial energy (newborn agent)	2
Energy to replicate (threshold)	10
Cost of replication (parent agent)	8
Base survival cost (per iteration)	0.01
Signaling cost (per intensity signal and iteration)	0.001
Range of signal intensity	[0; 1]
Range of neural network (NN) weights	[−1; 1]
Ratio of genes per NN weight	1
Number of weights per genotype	290
Gene mutation rate	0.05
Gene mutation maximum offset (uniform)	0.05

Presented in this table are the values of the key parameters used in the simulations.

## Results

### Emergence of swarming

**Fig 3:**
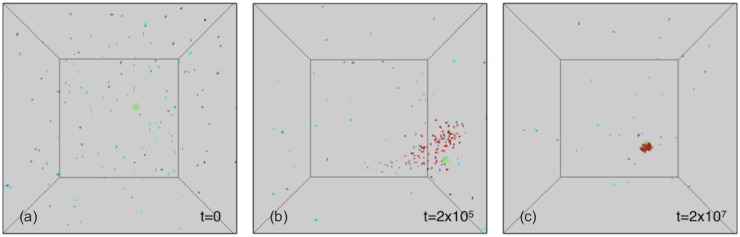
Visualization of the three successive phases in the training procedure (from left to right: *t* = 0, *t* = 2 ⋅ 10^5^, *t* = 2 ⋅ 10^7^) in a typical run. The simulation is with 200 initial agents and a single resource spot. At the start of the simulation the agents have a random motion (a), then progressively come to coordinate in a dynamic flock (b), and eventually cluster more and more closely to the goal towards the end of the simulation (c). The agents’ colors represent the signal they are producing, ranging from 0 (blue) to 1 (red). The goal location is represented as a green sphere on the visualization.

Agents are observed coordinating together in clustered groups. As shown in Fig 3 (top) the simulation goes through three distinct phases. In the first one, agents wander in an apparently random way across the space. During the second phase, the agents progressively cluster into a rapidly changing shape, reminiscent of animal flocks (as mentioned in the introduction, swarming can take multiple forms depending on the situation and/or the species, and is reminiscent of the swarming of mosquitoes or midges in this case). In the third phase, towards the end of the simulation, the flocks get closer and closer to the goal, forming a compact ball around it. Although results with one goal are presented in the paper, same behaviors were observed in the case of two or more resource spots.

**Fig 4:**
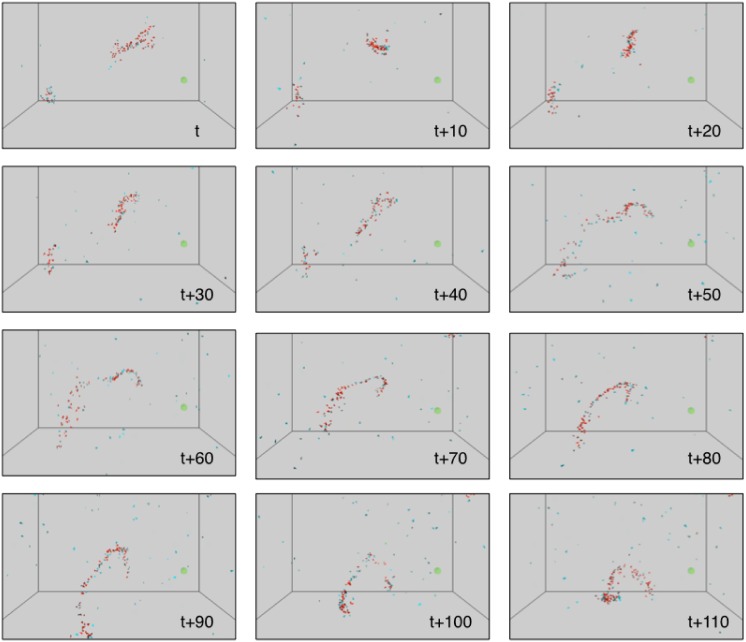
Visualization of the swarming behavior occurring in the second phase of the simulation. The figure represents consecutive shots each 10 iterations apart in the simulation. The observed behavior shows agents flocking in dynamic clusters, rapidly changing shape.

Fig 4 shows more in detail the swarming behavior taking place in the second phase. The agents coordinate in a dynamic, quickly changing shape, continuously extending and compressing, while each individual is executing fast paced rotations on itself. Note that this fast rotation seems to be needed to evolve swarming, as all trials with slower rotation settings never achieved this kind of dynamics. A fast rotation allows indeed each agent to react faster to the environment, as each turn making one sensor face a particular direction allows a reaction to the signals coming from that direction. The faster the rotation, the more the information gathered by the agent about its surroundings is balanced for every direction. The agents are loosely coupled, and one regularly notices some agents reaching the border of a swarm cluster, leaving the group, and ending up coming back in the heart of the swarm.

In spite of the agents needing to pay a cost for signaling (cf. description of the model above), we observe the signal to remain mostly between 0.2 and 0.5 during the whole experiment (in the case with signaling activated).

### Neighborhood

**Fig 5:**
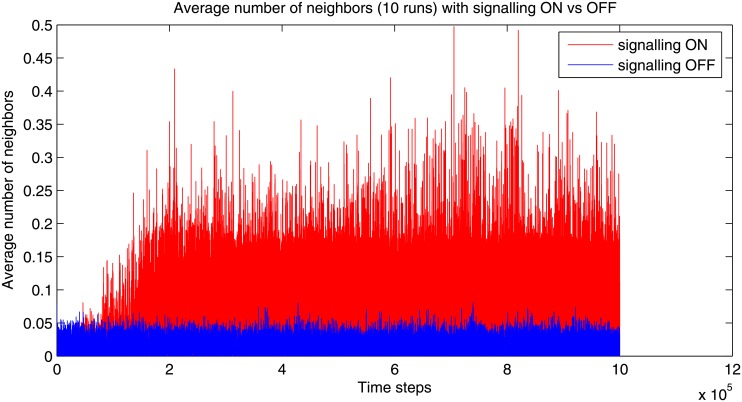
Comparison of the average number of neighbors (average over 10 runs, with 10^6^ iterations) in the case signaling is turned on versus off.

We choose to measure swarming behavior in agents by looking at the average number of neighbors within a radius of 100 distance around each agent. Fig 5 shows the evolution of the average number of neighbors, over 10 different runs, respectively with signaling turned on and off. A much higher value is reached around time step 10^5^ in the signaling case, while the value remains for the silent control. The swarming emerges only with the signaling switched on, and as soon as the signaling is silenced, the agents rapidly stop their swarming behavior and start wandering randomly in space.

We also want to measure the influence of each agent on its neighborhood, and vice versa. To do so, we use a measure of information transfer, to detect asymmetry in the interaction of subsystems [38]. The measure is to be applied on the time series of recorded states for different agents, and is based upon Shannon entropy. In the following, we first introduce the measure, then explain how we use it on our simulations.

### Transfer entropy measure

Shannon entropy *H* represents the uncertainty of a variable [39]. For a probability *p*(*x*), where *x* is the state of each agent (in our case we choose the instantaneous velocity), it is defined by:

$$\begin{array}{c} H(X)=-\sum \;p(x)log\;p(x) \end{array}$$ (7)

The mutual information *I* between two variables *X* and *Y* can be expressed as:

$$\begin{array}{c} I(X,Y)=H(Y)-H(Y|X) \end{array}$$ (8)

where *H*(*Y*) is the uncertainty of *Y* and *H*(*Y*|*X*) is the uncertainty of *Y* knowing *X*. The direction of causality is difficult to detect with mutual information, because of the symmetry: *M*(*X*, *Y*) = *MI*(*Y*, *X*) [40].

The transfer entropy from a time series *X* to another time series *Y* is a measure of the amount of directed transfer of information from *X* to *Y*. It is formally defined as the amount of uncertainty reduced in future values of *Y* by knowing a time window of *h* past values of *X*. This is written as follows:

$$\begin{array}{c} T_{X\to Y}=H\left(Y_{t}|Y_{t-1+d:t-h}\right)-H\left(Y_{t}|Y_{t-1+d:t-h},X_{t-1+d:t-h}\right) \end{array}$$ (9)

where *X*_*t*−1: *t*−*h*_ and *Y*_*t*−1: *t*−*h*_ are the past histories of length *h* for respectively *X* and *Y*, i.e. the *h* previous states counted backwards from the state at time *t*−1, and *d* is the time delay.

### Information flows in simulations

In our case, as the processes are the agent’s velocities, and thus they take values in $R^{3}$, we based our calculations on generalizations to multivariate and continuous variables as proposed in [40–43].

To study the impact of each agent on its neighborhood, the *inward* average transfer entropy on agent’s velocities is computed between each neighbor within a distance of 100.0 and the agent itself:

$$\begin{array}{c} T_{neighborhood\to self}=\sum_{i\in agents}\sum_{d(j,i)<r}T_{j\to i} \end{array}$$ (10)

**Fig 6:**
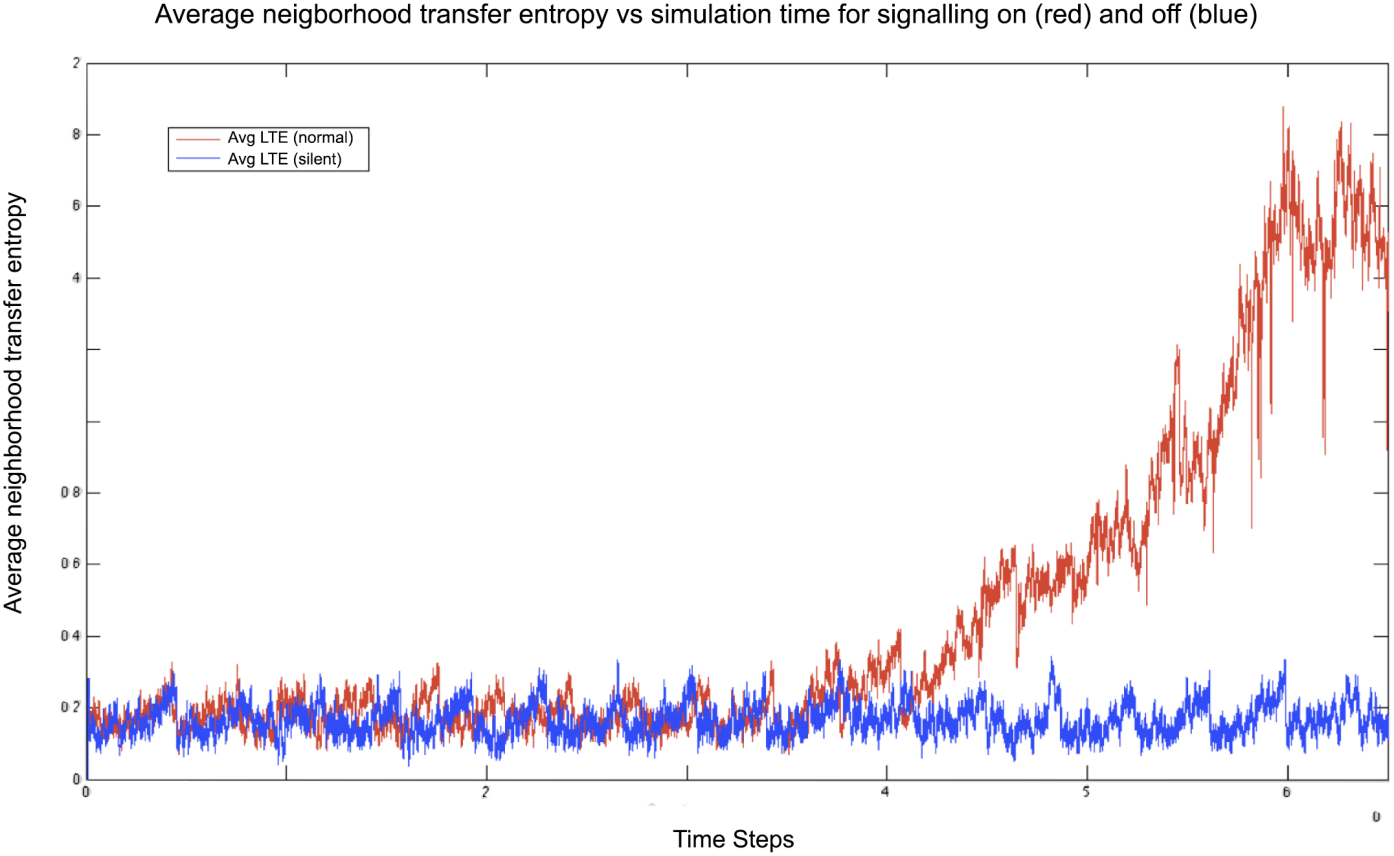
Plot of the average *inward* neighborhood transfer entropy for signaling switched on (red curve) and off (blue curve). The inward neighborhood transfer entropy captures how much agents are “following” individuals located in their neighborhood at a given time step. The values rapidly take off on the regular simulation (with signaling switched on, see red curve), whereas they remain low for the silent control (with signaling off, see blue curve).

We will refer to this measure as inward neighborhood transfer entropy (NTE). This can be considered a measure of how much the agents are “following” their neighborhood at a given time step. The values rapidly take off on the regular simulation (with signaling switched on), while they remain low for the silent control, as we can see for example in Fig 6.

**Fig 7:**
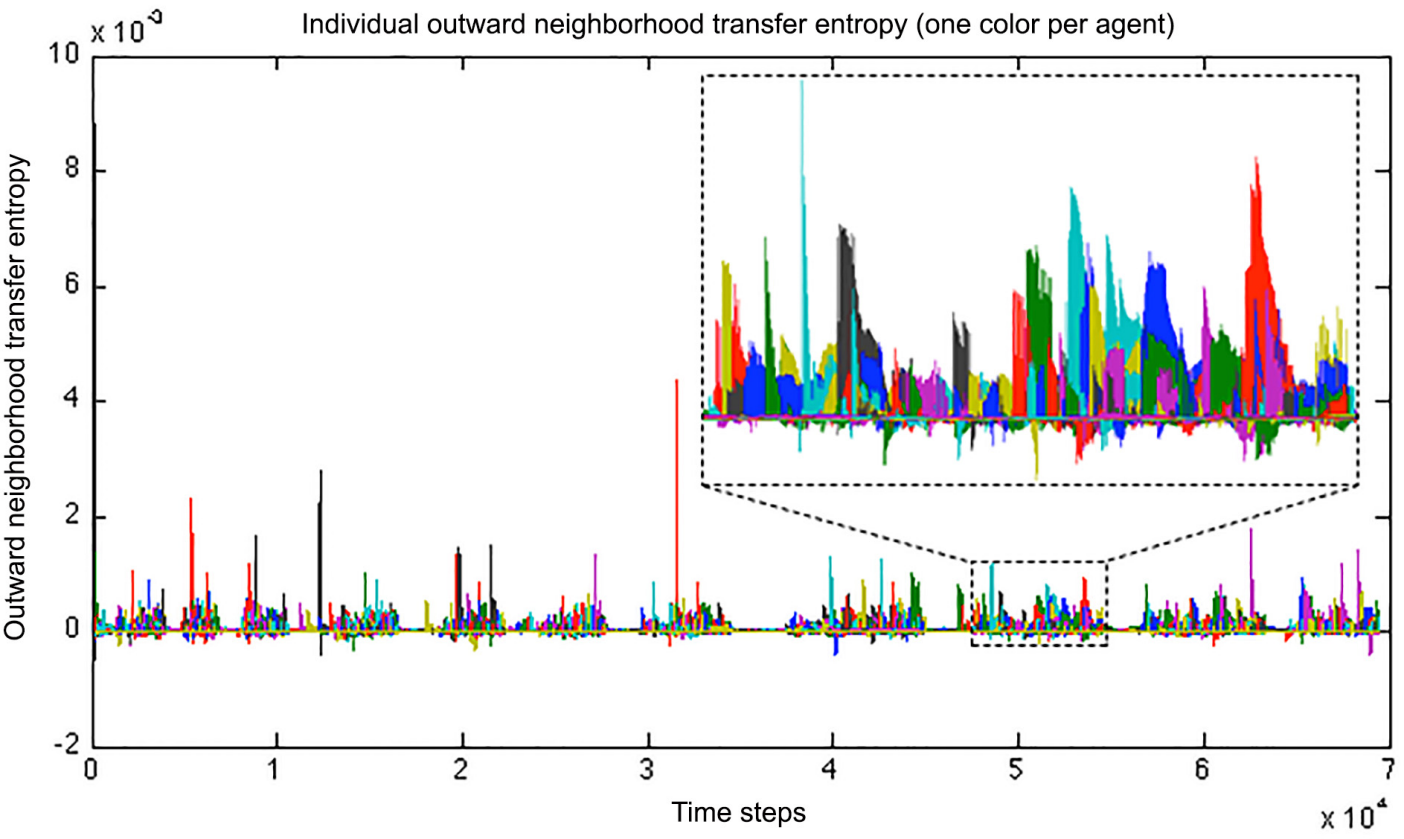
Plot of the individual *outward* neighborhood transfer entropy (NTE), aiming to capture the change in leadership. Detail from 4.75 10^4^ to 5.45 10^4^ time steps. The plot represents the average transfer entropy from an agent to its neighbors, capturing the presence of local leaders in the swarming clusters. Each color corresponds to a distinct agent. A succession of bursts is observed, each corresponding to a different agent, indicating a continual change of leadership in the swarm.

Similarly, we can calculate the *outward* neighborhood transfer entropy (i.e. the average transfer entropy from an agent to its neighbors). We may look at the evolution of this value through the simulation, in an attempt to capture the apparition of local leaders in the swarm clusters. Even though the notion of leadership is hard to define, the study of the flow of information is essential in the study of swarms. The single individuals’ *outward* NTE shows a succession of bursts coming every time from different agents, as illustrated in Fig 7. This frequent switching of the origin of information flow can be interpreted as a continual change of leadership in the swarm. The agents tend to follow a small number of agents, but this subset of leaders is not fixed over time.

**Fig 8:**
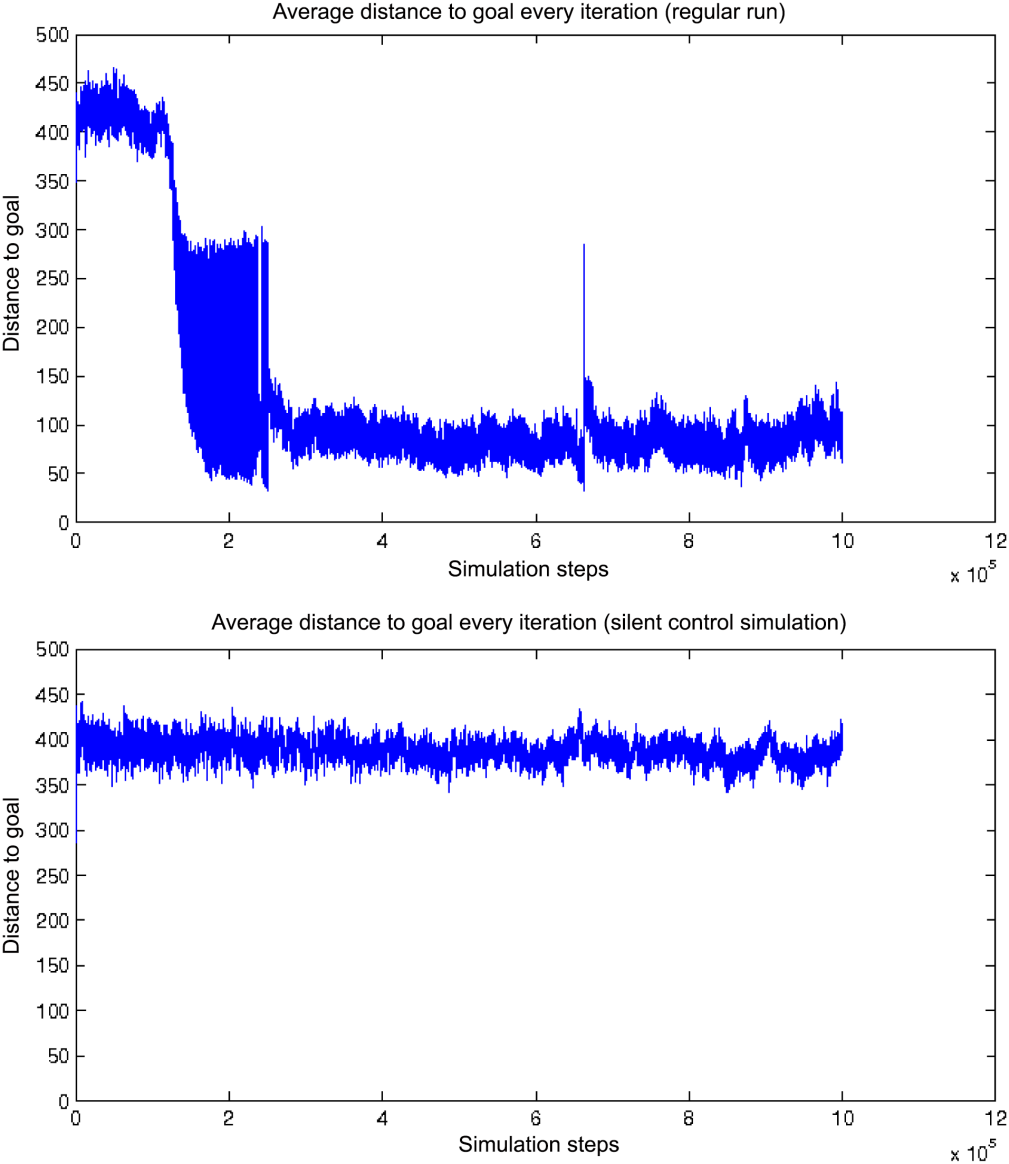
Average distance of agents to the goal with signaling (top) and a control run with signaling switched off (bottom). The average distance to the goal decreases between time step 10^5^ and time step 2 × 10^5^, the agents eventually getting as close as 50 units away from the goal on average. In the same conditions, the silenced control experiment results in agents constantly remaining around 400 units away from the goal in average.

On the upper graph in Fig 8, between iteration 10^5^ and 2 × 10^5^, we see the average distance to the goal drop to values oscillating between roughly 50 and 300, that is the best agents reach 50 units away from the goal, while other agents remain about 300 units away. On the control experiment graph (Fig 8, bottom), we observe that the distance to the goal remains around 400.

Swarming, allowed by the signaling behavior, allows agents to stick close to each other. That ability allows for a winning strategy in the case when some agents already are successful at remaining close to a resource area. Swarming may also help agents find goals in the fact that they constitute an efficient searching pattern. Whilst an agent alone is subject to basic dynamics making it spatially drift away, a bunch of agents is more able to stick to a goal area once it finds it, since natural selection will increase the density of surviving agents around those areas. In the control runs without signaling, it is observed that the agents, unable to form swarms, do not manage to gather around the goal in the same way as when the signaling is active.

### Controller response

**Fig 9:**
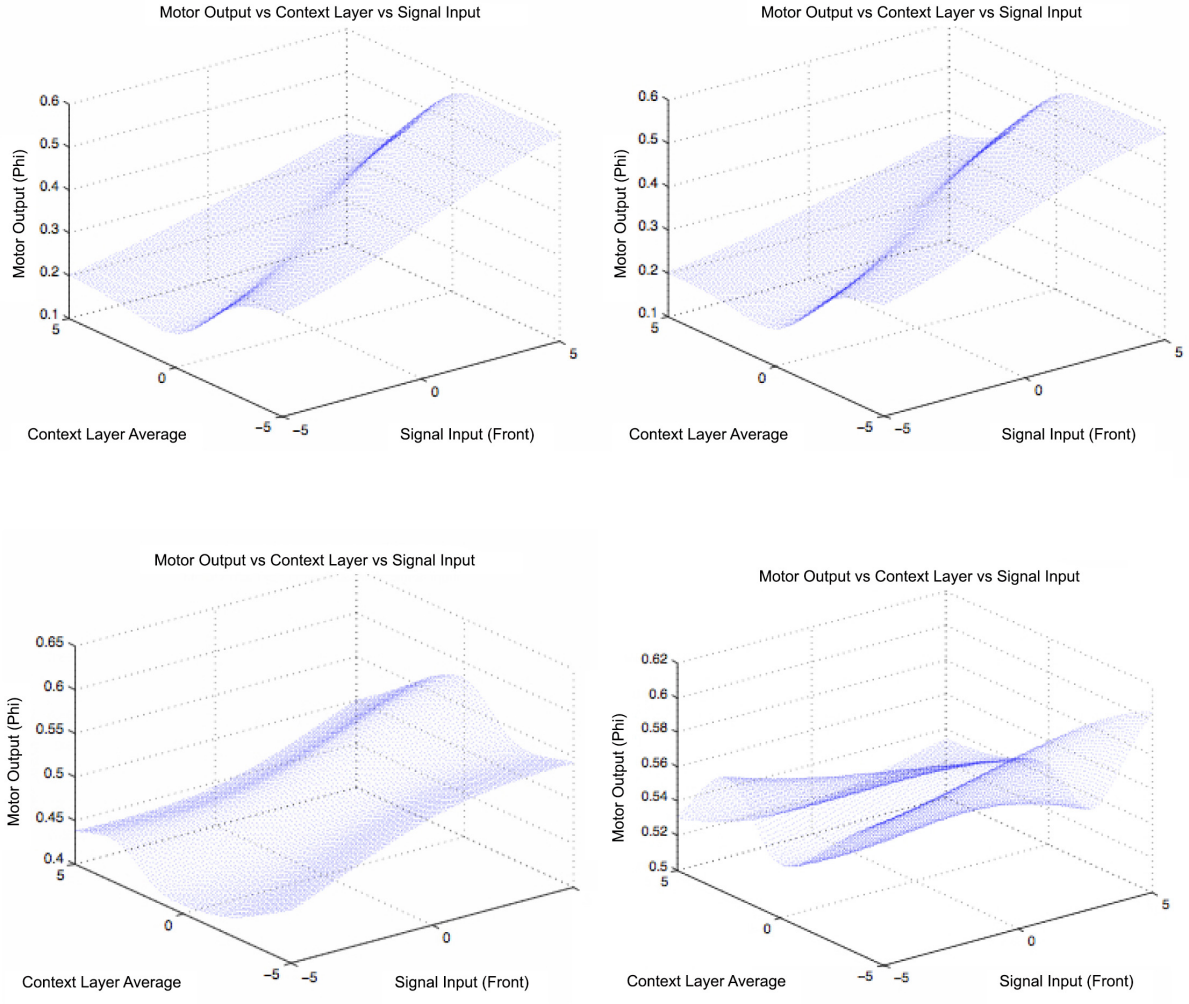
Plots of evolved agents’ motor responses to a range of value in input and context neurons. The three axes represent signal input average values (right horizontal axis), context unit average level (left horizontal axis), and average motor responses (vertical axis). The *top* two graphs correspond to the neural controllers of swarming agents, and the *bottom* ones correspond to non-swarming ones’.

Once the training step is over, the neural networks of all agents are tested, and swarming agents are compared against non-swarming ones. In particular, we plotted on Fig 9 for a range of input values for the front sensor, the resulting motor output *o*_1_ (controlling the rotation of the velocity vector about the *y* axis) and the average activation of neurons in the context layer. In practice, this is equivalent to plotting the rotation rate against local agent density and against “memory activation”. We observed that characteristic shapes for the curve obtained with swarming agents presented a similarity (see Fig 9, top), and differed from the patterns of non-swarming agents (see Fig 9, bottom) which were also more diverse.

In swarming individuals’ neural networks, patterns were observed leading to higher motor output responses in the case of higher signal inputs. This is characteristic of almost every swarming individual, whereas non-swarming agents present a wide range of response functions. A higher motor response allows the agent to slow down its course across the map by executing quick rotations around itself, therefore keeping its position nearly unchanged. If this behavior is adopted in the cases in which the signal is high, that is in the presence of signaling agents, the agent is able to remain close to them. Steeper slopes in the response curves in Fig 9 may consequently lead to more compact swarming patterns. Those swarming patterns are observed after longer simulation times.

This dual pattern of motion is reminiscent of species such as Chlamydomonas reinhardtii [44]. This single-cell green alga moves by beating its two flagella either synchronously or asynchronously, leading respectively to spiraling along a rectilinear trajectory or rotating around a fixed axis.

### Signaling

On the one hand signaling having a cost in energy, one expects it to be selected against in the long run since it lowers the survival chances of the individual. However, if the signaling behavior is beneficial to the agents, it may be selected for. But agents that do not signal may profit from the other agents’ signals and still swarm together. A value close to zero for the signal saves them a proportional cost of energy in signaling, hypothetically allowing those freeriders to spend less energy and eventually take over the living population.

In order to study the agent’s choice of signaling over remaining silent, we examine the effect of artificially introducing silent agents in the population. To that purpose, during a run at the end of its training step, 5 agents are picked at random in the population, and their genotype is modified such that the value of the signal they produce becomes zero. Indeed, the values in each agent’s genotype encodes directly the weights of its artificial neural network. In order for the rest of the controller response to be identical, the only weights being changed are the ones of the connections to the signal output (*O*_3_ on the diagram in Fig 2).

**Fig 10:**
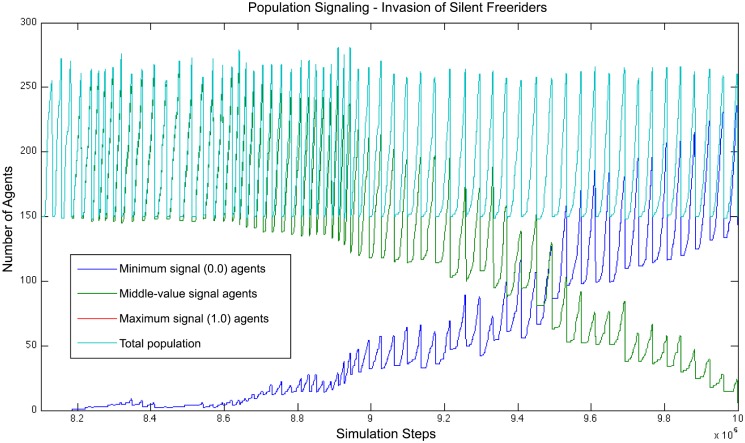
Invasion of freeriders resulting from the introduction of 5 silent individuals in the population. About 200*k* iterations after their introduction, the 5 freeriders have replicated and taken over the whole population.

As a result, the modified (silent) agents take over the population, slowly replacing the signaling agents. As the signaling agents progressively disappear from the population (cf. Fig 10), so does the clustering behavior. About 200*k* iterations after the introduction of the freeriders, the whole population has been replaced by freeriders and the swarming behavior has stopped. This confirms silent freeriding as an advantageous behavior when a part of the population is already swarming, however leading to the advantageous swarming trait being eradicated from the population after a certain time. If we then let the simulation run, we observe that the signaling behavior will end up evolving again, after a variable time depending on the seed. Although it is not in the scope of this paper, further study could focus on how certain areas of the genetic space will influence the probability to evolve it again in a given simulation time.

**Fig 11:**
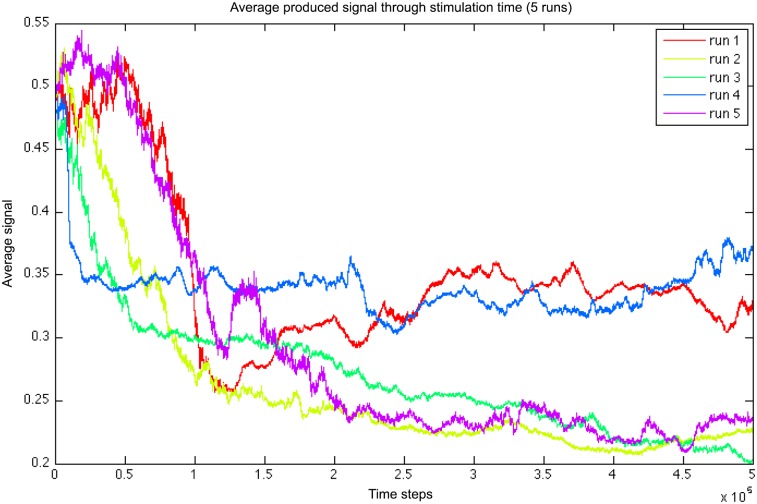
Average signal intensity over the population versus evolutionary time (5 runs).

If there is an evolutionary advantage to swarming, and if that behavior relies on signaling, the absence of signaling directly reduces the swarm’s fitness. This is not the case however if the change in signaling intensity occurs progressively, slowly leading to a lower, cost-efficient signaling, while swarming is still maintained. We observe this effect of gradual decrease in average signal at Fig 11.

The reason why in the original simulation, the whole population is not taken over by freeriders is that smaller changes to the genotype, smoothly making the signaling lower, is not going to invade the whole population in the same way. Indeed, in that case, the population is replaced by individuals signaling gradually less. In our evolutionary scheme, the genes controlling the signal intensity do not drop quickly enough to make the signal intensity drop to zero. Instead, a swarm will have its signal intensity drop down to a point where it is still fit enough to be selected for, against other groups, which illustrates a limited case of group selection. A detailed analysis of these dynamics is however not in the scope of this paper.

### Genotypic diversity

**Fig 12:**
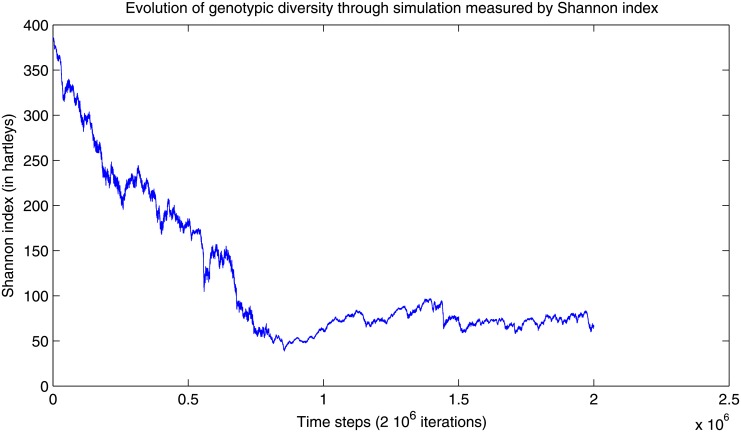
Genotypic diversity measured by Shannon’s information entropy. The information entropy measures the variety in the measure progressively decreases during the simulation, until it reaches a minimal value of 50 hartleys (information unit corresponding to a base 10 logarithm) around the millionth iteration, then restarts to increase slowly.

The decisions of each agent are defined by the parameters describing its neural controller, which are encoded directly in each agent’s genotype. That genotype is evolved via random mutation and selection in the setup environment. In order to study the variety of the genotypes through the simulation, the average Shannon entropy [39] is calculated over the whole population using:

$$\begin{array}{c} H=-\sum_{i=1}^{n}p_{i}\;log\;p_{i} \end{array}$$ (11)

where *p*_*i*_ is the frequency of genotype *i*. The frequency is the proportion of a particular combination of genes among all combinations being considered. The value of *H* ranges from 0 if all the genotypes are similar, to *log* *n* for evenly distributed genotypes, i.e. $\forall \;i\;p_{i}=\frac{1}{n}$. It should be noted that as every allele (i.e. value in the genotype vector) takes a floating point value, we discretize those in 5 classes per value to allow for a measure of frequencies. *H* is used as a measure of genotypic variety and plotted against simulation time (Fig 12). The measure progressively decreases during the simulation, until it reaches a minimal value of 50 hartleys (information unit corresponding to a base 10 logarithm) around the millionth iteration, before restarting to increase, with a moderate slope. The fast drop in diversity is explained by a strong selection for swarming individuals in the first stage of the simulation. Once the advantageous behavior is reached, a genetic drift can be expected, resulting in genetic drift and reduced selection, as will be discussed further below.

### Phylogeny

**Fig 13:**
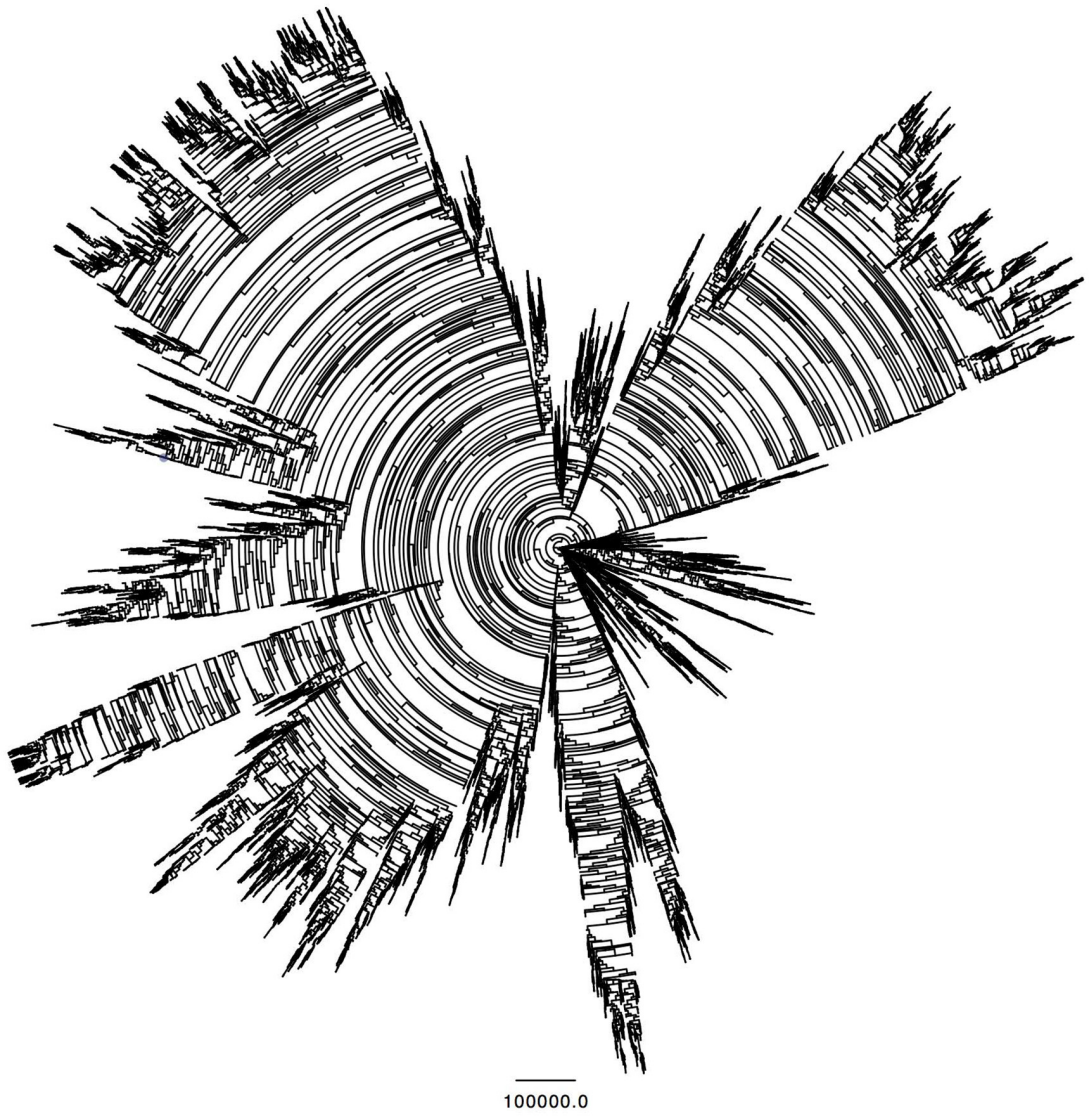
Phylogenetic tree of agents created during a run. The center corresponds to the start of the simulation. Each branch represents an agent, and every fork corresponds to a reproduction process.

The heterogeneity of the population is visualized on the phylogenetic tree at Fig 13. At the center of the graph is the root of the tree, which corresponds to time zero of the simulation, from which start the 200 initial branches, i.e. initial agents. As those branches progress outward, they create ramifications that represent the descendance of each agent. The time step scale is preserved, and the segment drawn below serves as a reference for 10^5^ iterations. Every fork corresponds to a newborn agent. The parent forks counterclockwise, and the newborn forks clockwise. Therefore, every “fork burst” corresponds to a period of high fitness for the concerned agents.

**Fig 14:**
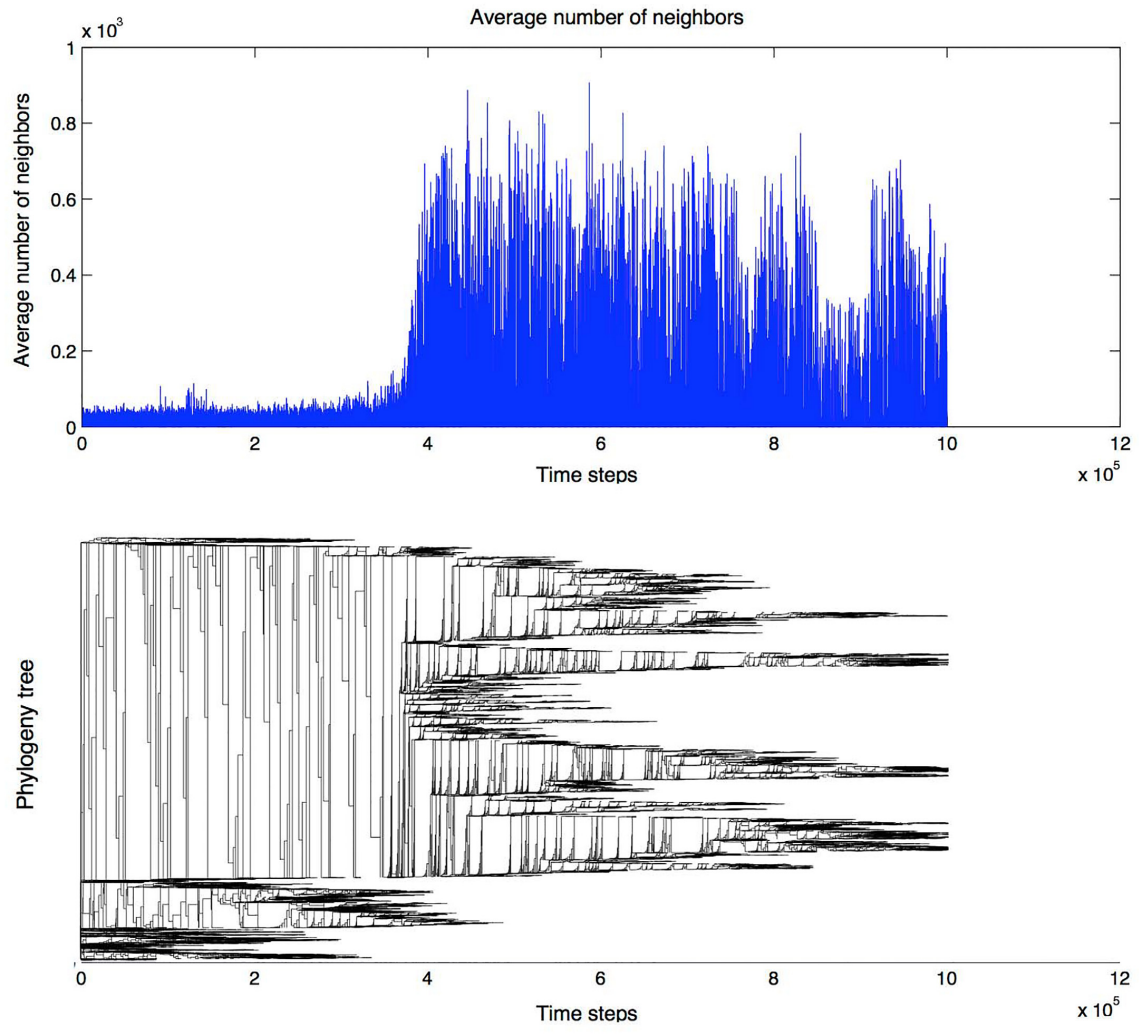
Top plot: average number of neighbors during a single run. Bottom plot: agents phylogeny for the same run. The roots are on the left, and each bifurcation represents a newborn agent. The two plots show the progression of the average swarming in the population, indicated by the average number of neighbors through the simulation, compared with a horizontal representation of the phylogenetic tree. Around iteration 400*k*, when the neighborhood becomes denser, the selection on agents’ ability to swarm together is apparently relaxed due to the signaling pattern being largely spread. This leads to higher heterogeneity, as can be seen on the upper plot, with numerous genetic branches forming towards the end of the simulation.

In Fig 14, one can observe another phylogenetic tree, represented horizontally in order to compare it to the average number of neighbors throughout the simulation. The neighborhood becomes denser around iteration 400*k*, showing a higher portion of swarming agents. This leads to a firstly strong selection of the agents able to swarm together over the other individuals, a selection that is soon relaxed due to the signaling pattern being largely spread, resulting in a heterogeneous population, as we can see on the upper plot, with numerous branches towards the end of the simulation.

The phylogenetic tree shows some heterogeneity, and the average number of neighbors is a measure of swarming in the population. The swarming takes off around iteration 400k, where there seems to be a genetic drift, but the signaling helps agents form and maintain swarms.

To study further the relationship between heterogeneity and swarming, we classify the set of all the generated genotypes with a principal component analysis or PCA [45]. In practice, we operate an orthogonal transformation to convert the set of weights in every genotype into values of linearly uncorrelated variables called principal components, in such a way that the first principal component *PC*1 has the highest possible variance, and the second component *PC*2 has the highest variance possible while remaining uncorrelated with *PC*1.

**Fig 15:**
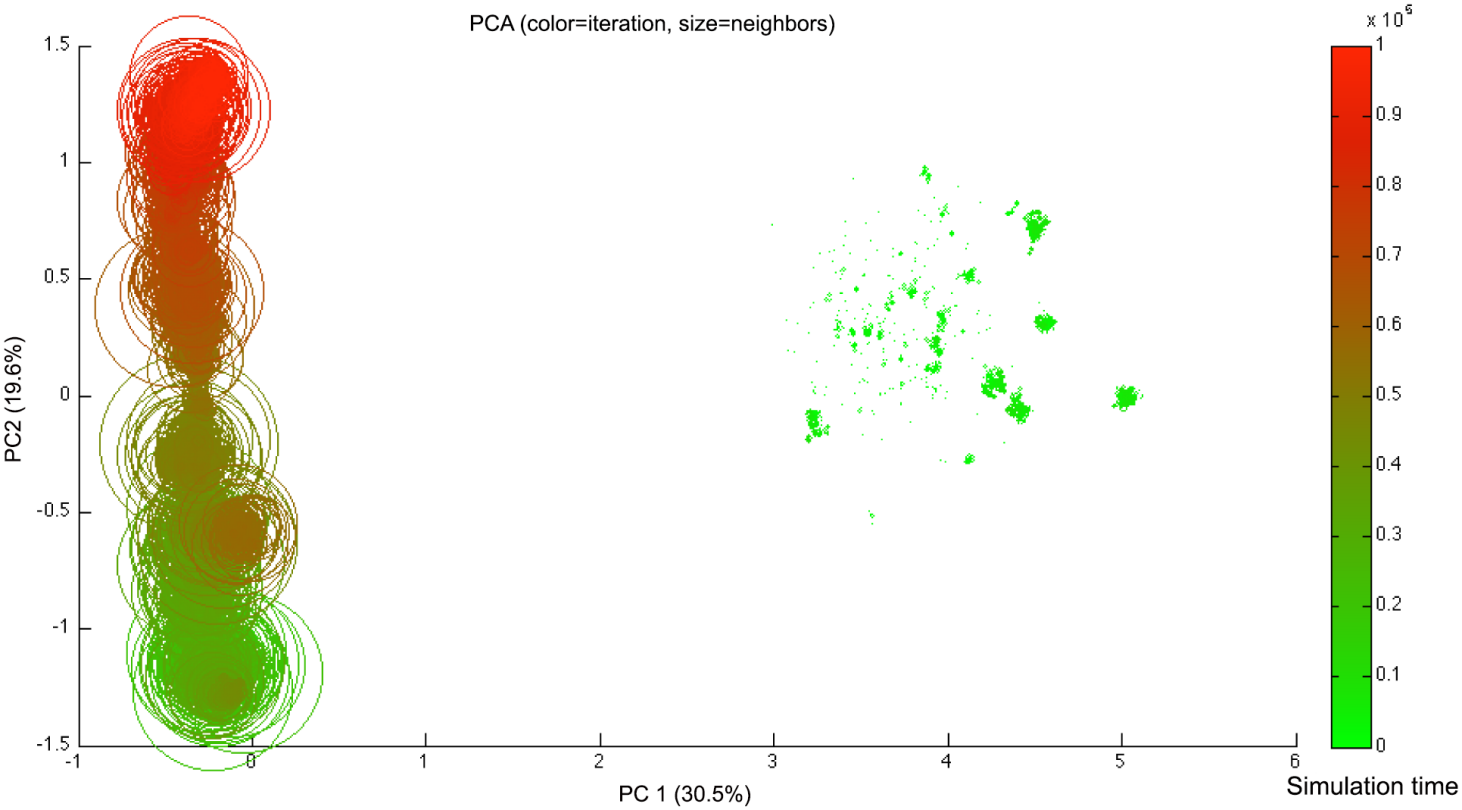
Biplot of the two principal components of a PCA on the genotypes of all agents of a typical run, over one million iterations. Each circle represents one agent’s genotype, the diameter representing the average number of neighbors around the agent over its lifetime, and the color showing its time of death ranging from bright green (at time step 0, early in the simulation) to red (at time step 10^6^, when the simulation approaches one million iterations).

In Fig 15, we observe a large cluster on the left of the plot for *PC*1 ∈ [−1; 0], and a series of smaller clusters on the right for *PC*1 ∈ [3; 5]. The genotypes in the early stages of the simulation belong to the right clusters, but get to the left cluster later on, reaching a higher number of neighbors.

The classification shows a difference between early and late stages in terms of genotypic encoding of behavior. The genotypes are first observed to reach the left side cluster on the biplot, which differs in terms of the component *PC*1. It also corresponds to a more intensive swarming, as shown by the individuals’ average number of neighbors. The agents then remain in that cluster of values for the rest of the simulation. The timing of that first change corresponds to the first peak in number of neighbors, which is an index for the emergence of swarming. The agents’ genotypes then seem to evolve only slowly in terms of *PC*2, until they reach the last and highest peak in number of neighbors. This very slow and erratic increase is correlated with the slow drop in the radius of the clusters, due to steeper slopes of controller responses (see Fig 9) on long runs.

## Discussion

In this work we have shown that swarming behavior can emerge from a communication system in a resource gathering task. We implemented a three-dimensional agent-based model with an asynchronous evolution through mutation and selection. The results show that from decentralized leader-follower interactions, a population of agents can evolve collective motion, in turn improving its fitness by reaching invisible target areas. The trajectories are reminiscent of swarms of cells such as Chlamydomonas, which alternate synchronous and asynchronous swimming using their two flagella [44].

However, the intention in this paper is not to imitate perfectly animal swarms in nature, but rather study how an approach from evolutionary robotics, with neural networks evolved via an asynchronous genetic algorithm, could lead to the emergence of such collective behavior. The obtained swarming genuinely corresponds to the conditions of the embodiment given to the agents in our simulation, which models a species unable to directly perceive its fitness landscape. The absence of sensors to detect gradients of resources forces the agents to find different ways to optimize their survival, thus letting them evolve a swarming behavior. This paper can as well be read through the lens of the evolutionary metaphor for a species performing an uninformed search for optimal ecological niches. In that case, the three-dimensional space becomes just an abstract representation of the role and position of the species in its environment, i.e. how it feeds, survives and reproduces.

Our results represent an improvement on models that use hard-coded rules to simulate swarming. Here, the behavior is evolved with a simplistic model, based on each individual’s computation, which truly only perceive and act on their direct neighborhood, as opposed to a global process controlling all agents such as the boids introduced by [17]. The model also improves on previous research in the sense that agents naturally switch leadership and followership by exchanging information over a very limited channel of communication. Indeed, it does not rely on any explicit information from leaders like in [46, 20] and [21], nor does it even impose any explicit leader-follower relationship beforehand, letting simply the leader-follower dynamics emerge and self-organize. The swarming model presented in this paper offers a simple, general approach to the emergence of swarming behavior once approached via the boids rules. Other studies such as [26] and [25] have approached swarming without an explicit fitness. Although their simulation models a predator-prey ecology, the type of swarming they obtain from simple pressures is globally similar to the one in this study. However, a crucial difference is that our model presents the advantage of a simpler self-organized system merely based on resource finding and signaling/sensing. Our results also show the advantage of swarming for resource finding (it is only through swarming, enabled by signaling behavior, that agents are able to reach and remain around the goal areas), comparable to the advantages of particle swarm optimizations [23], here emerging in a model with a simplistic set of conditions. Contrary to previous work studying environmental gradients climbing [27], the motion leading to swarming was here not a coded behavior but rather evolved through generations of simulated agents.

In the simulations, the agents progressively evolve the ability to flock using communication to perform a foraging task. We observe a dynamical swarming behavior, including coupling/decoupling phases between agents, allowed by the only interaction at their disposal, that is signaling. Eventually, agents come to react to their neighbors’ signals, which is the only information they can use to improve their foraging. This can lead them to either head towards or move away from each other. While moving away from each other has no special effect, moving towards each other, on the contrary, leads to swarming. Flocking with each other may make agents slow down their pace, which for some of them may keep them closer to a food resource. This creates a beneficial feedback loop, since the fitness brought to the agents will allow them to reproduce faster, and eventually spread the behavior within the whole population. In this scenario, agents do not need extremely complex learning to swarm and eventually get more easily to the resource, but rather rely on group dynamics emerging from their communication system to increase inertia and remain close to goal areas.

The swarming constitutes an efficient dynamic search pattern, that improves the group’s chances to find resource. Indeed, the formation of a cluster slows agents down, allowing them to get more resource whenever they reach a favorable spot, which gives the agents more chance to replicate. Also, as a cluster, the agents are less likely to be wiped off because of some individuals drifting away from the resource. As long as the small mistakes are still corrected as newcomers join the group, the swarm can be sustained. Importantly, such dynamic swarming pattern is only observed in the transient phase, when the agents are still moving across the map, i.e. before any group has stabilized its position around a fixed resource area, thus making the swarms more and more compact.

Lastly, we provide five ending remarks about the swarming dynamics uncovered in this paper:

The simulation allows for strong genetic heterogeneity due to the asynchronous reproduction schema, as could be visualized in the phylogenetic tree. Such genetic, and thus behavioral heterogeneity may suppress swarming but the evolved signaling helps the population to form and keep swarming. The simulations do not exhibit strong selection pressures to adopt specific behavior apart from the use of signaling. Without high homogeneity in the population, the signaling alone allows for interaction dynamics sufficient to form swarms, which proves in turn to be beneficial to get extra fitness, as mentioned above. The results suggest that by coordinating in clusters, the agents enter an evolutionary neutral space [47], where little selection is applied to their genotypes. The formation of swarms acts as a shield on the selection process, as a consequence allowing for the genotypes to drift. This relaxation of selection can be compared to a niche construction, in which the system is ready to adapt to further optimizations to the surrounding environment. This may be examined in further research by the addition of a secondary task.
In the presented model, the population of genotypes progressively reaches the part of the behavioral search space that corresponds to swarming, as it helps agents achieve a higher fitness. The behavioral transition between non-swarming and swarming happens relatively abruptly, and can be caused by either the individual behavior improving enough or the population dynamical state satisfying certain conditions, or a combination of both. The latter one is highlighted by the variable amount of time necessary before swarms can reform after the positions have been randomized, thus illustrating the concept of collective memory in groups of self-propelled individuals. Indeed, although one agent’s behavior is dictated by its genotype, the swarming also depends on the collective state of the neighborhood. Couzin et al. [4] brought to attention that even for identical individual behaviors, the previous history of a group structure can change its dynamics. In the light of that fact, reaching the neutral space relies on more than just the individual’s genetic heritage.
The swarming behavior that our model demonstrates in phase 2, before it starts overfitting in phase 3, may be an example of emergence of criticality in living systems [48]. The coevolutionary and coadaptive mechanisms by which populations of agents converge to be almost critical, in the process of interacting together and forming a collective entity, is typically demonstrated in phase 2, showing agents reaching this critical evolutionary solution in their striving to cope in a complex environment.
The leadership in the simulated swarms is not fixed, but temporally changing, as shown by our measures of the transfer entropy among agents. This dynamic alternation of leaders may change depending on the swarm approaching or getting away from food sites. The initiation of leadership is an interesting open question for simulating swarming behaviors, which should be examined in further work.
The phenomenon of freeriding, observed when artificially introducing silent individuals, is comparable to a tragedy of the commons (ToC) or an evolutionary suicide, in which an evolved selfish behavior can harm the whole population’s survival [49, 50]. This effect, here provoked artificially, is however unlikely to happen in our setup, as the decrease in produced signal intensity would progressively result in an inefficient performance, with a smooth decrease of fitness over the search space. The ToC has better chances to arise in a setup with a larger map, in which parts of the population can be isolated for a longer time, leading to different populations evolving separately, until they meet again and confront their behaviors.
